# Immunosuppressive therapy for progressive idiopathic membranous nephropathy: a cost-effectiveness analysis in China

**DOI:** 10.1186/s12913-023-09365-z

**Published:** 2023-04-12

**Authors:** Wanyi Xu, Zhiqi Zhang, Dandan Li, Wendi Dai, Chen Pan, Mingxing Guo, Ying Zhao, Xiangli Cui

**Affiliations:** 1 Department of Pharmacy, Beijing Friendship Hospital, Capital Medical University, Yong-an Road, Xi-Cheng District, Beijing, 100050 PR China; 2 School of Pharmacy, Capital Medical University, Beijing, PR China; 3 Department of Nephrology, Beijing Friendship Hospital, Capital Medical University, Beijing, PR China

**Keywords:** Idiopathic membranous nephropathy, Immunosuppressive therapy, Cost-effectiveness

## Abstract

**Background:**

This study aims to evaluate the cost-effectiveness of immunosuppressive therapy for patients with progressive idiopathic membranous nephropathy (IMN) from the Chinese healthcare system perspective.

**Methods:**

To estimate the cost-effectiveness of four regimens namely cyclophosphamide, cyclosporine, rituximab and tacrolimus-rituximab in treatment of IMN recommended by the updated *Kidney Disease: Improving Global Outcomes* (KDIGO) guideline 2021, a Markov model with five discrete states (active disease, remission, dialysis, kidney transplant and death) based on IMN patients aged 50 or above over a 30-years time horizon was constructed. Total costs were imputed from the Chinese healthcare system perspective, and health outcomes were converted into quality-adjusted life years (QALYs). The incremental cost-effectiveness ratio (ICER) was used to describe the results. The willingness-to-pay (WTP) threshold was set at $12,044 (China’s 2021 Gross Domestic Product per capita). Sensitivity analyses were performed to test the uncertainties of the results.

**Result:**

Compared with cyclophosphamide, both cyclosporine (incremental cost $28,337.09, incremental QALY-1.63) and tacrolimus-rituximab (incremental cost $28,324.13, incremental QALY -0.46) were considered at strictly dominated for their negative values in QALYs, and the ICER value of rituximab was positive (incremental cost $9,162.19, incremental QALY 0.44). Since the ICER of rituximab exceeds the pre-determined threshold, cyclophosphamide was likely to be the best choice for the treatment of IMN within the acceptable threshold range. The results of the sensitivity analysis revealed that the model outcome was mostly affected by the probability of remission in rituximab. In a probabilistic sensitivity analysis, cyclophosphamide had 62.4% probability of being cost-effective compared with other regimens when the WTP was $12,044 per QALY. When WTP exceeded $18,300, rituximab was more cost-effective than cyclophosphamide.

**Conclusion:**

Compared with cyclosporine, rituximab and tacrolimus-rituximab, our model results indicated that cyclophosphamide represented the most cost-effective regimen for patients with progressive IMN in China.

**Supplementary Information:**

The online version contains supplementary material available at 10.1186/s12913-023-09365-z.

## Introduction

Idiopathic membranous nephropathy (IMN) is the most common type of primary glomerulonephritis and a leading cause of nephrotic syndrome in adults [1]. It is noteworthy that the incidence of IMN has increased markedly comparing with other types of glomerular diseases in recent years [2]. In China, it was reported that, over the 2011 ~ 2014 period, the annual incidence rate of IMN accounted for 24.1% of all kidney biopsy patients, which was nearly twice as much as the figure over 2003 ~ 2006 [3]. Approximately, 40% to 50% of IMN patients experience a spontaneous remission, the remaining patients would slowly progress to end-stage renal disease (ESRD) and die within 5 to 15 years since the onset of the disease [4]. Therefore, effective treatments are required for patients at moderate or high risk for progressive kidney injury to prevent the disease progression.

Cyclophosphamide and calcineurin inhibitors (CNIs) (cyclosporine and tacrolimus) are standard treatments for IMN. Despite their treatment effectiveness, both drugs are associated with frequent side effects and high possibility of relapse to active disease after drug withdrawal [5–7]. Recently, rituximab, a monoclonal antibody that targets the CD20 antigen expressed on the surface of pre-B and mature B-lymphocytes, has demonstrated promising effects with lower adverse reactions and better tolerance for IMN [8]. Hence, rituximab was recommended as the first-line treatment by the KDIGO guideline 2021 (*Kidney Disease: Improving Global Outcomes*) [9]. For patients with high risk of progression, addition of rituximab after 6 months of treatment with CNI is also advised as first-line treatment regimen, tacrolimus-rituximab regimen is a case in point. However, due to its high cost, this new therapy has imposed a heavy economic burden on both patients and the whole society with limited budget. A report reveals that the annual medication cost per-patient increased by 6.8 folds from $205 to $1,394 over the 2000 ~ 2012 period in Canada. The fast growth in incremental costs of CNIs and rituximab accounted for 94.5% of medication costs and were used by 44.6% of patients in 2013 comparing with 17.6% and 2.2% respectively in 2000 [10]. Therefore, for developing countries like China, it is essential to evaluate the financial impact of introducing immunosuppressive therapies on healthcare budget. The objective of this study was to develop a Markov model to evaluate the cost-effectiveness of cyclophosphamide, cyclosporine, rituximab and tacrolimus-rituximab from the Chinese healthcare system perspective.

## Materials and methods

### Patients and regimens

The target population for our economic analysis was patients diagnosed with IMN by renal biopsy at the onset age of 50 or above and at moderate and high risk of developing progressive kidney injury [11]. According to the recommendations of the KDIGO guideline 2021 and expert consensuses in China [9, 12, 13], four interventions selected for comparison were showed as follows: rituximab group, intravenous rituximab (375 mg/m^2^) on day 1 and day 15; cyclosporine group, oral cyclosporine (starting at a dose of 3.5 mg/kg/day for 12 months); tacrolimus-rituximab group, oral tacrolimus (0.05 mg/kg/day) to reach target blood levels of 5–7 ng/mL for 6 months and reduced by 25% per month until complete withdrawal at the ninth month. On day 180, patients received one dose of intravenous rituximab (375 mg/m^2^); cyclophosphamide group, a 6-months cyclic regimen with corticosteroids alternating with cyclophosphamide every other month. Patients received methylprednisolone in the first, the third and the fifth month (1 g cyclophosphamide intravenously on the first three days, followed by cyclophosphamide 0.5 mg/kg/day orally from day 4 to day 30). In the second, the fourth and the sixth month, patients received oral cyclophosphamide adjusted for age and renal function (1.0 mg/kg/day for 30 days). In this study, we assumed the average body weight of 60 kg and the body surface area of 1.6m^2^ [14]. Dose adjustment and the exchange between intervention strategies were excluded from this study. We assumed that all patients had full compliance under four therapy regimens in our study.

### Markov model

In this study, a Markov model was developed by using Tree Age Pro 2021 to predict the clinical events and outcomes of each progressive IMN patient over time under cyclophosphamide, cyclosporine, rituximab and tacrolimus-rituximab. The analysis employed the cost-effectiveness framework where the main measure of benefit is the quality-adjusted life years (QALYs) and with analysis outcomes represented by incremental cost-effectiveness ratios (ICERs) of cost per additional QALYs gained.

**Fig. 1:**
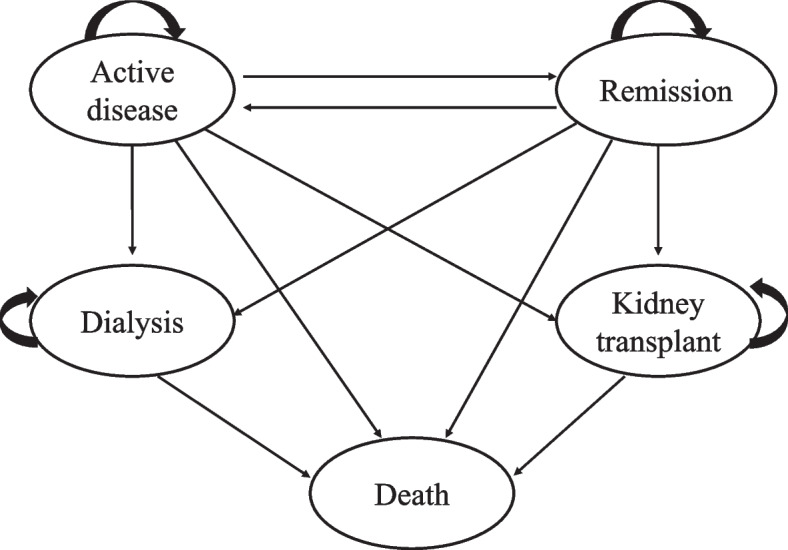
Markov structural model of health states with disease progression

The model included five health states according to the clinical development of progressive IMN: active disease, remission (partial or complete remission), dialysis, kidney transplant and death. Dialysis and kidney transplant were collectively known as ESRD. All patients were assumed to be in active disease before medical intervention. Afterwards, patients would stay in active state or progress to three other discrete states: remission, ESRD or death. In remission state, patients could remain in sustained remission, or experience relapse and return to the active state, or progress to the ESRD or death. Since patients in ESRD eventually need dialysis or kidney transplant, so we included both dialysis and kidney transplant as separate states instead of ESRD in the flow chart as shown in Fig. 1. The tree diagram of the Markov model was fully displayed in Supplementary Fig. S1. For patients in ESRD stage (either in dialysis state or in kidney transplant state), the patients would either maintain the current state or progress to death. In Markov model, patients transitioned between health states on one-year cycles over a lifetime horizon of 30 years. IMN is generally considered a disease of middle age with the median age of patients at diagnosis being 53 years [15]. Its average life expectancy is 76.34 years in China [16]. In this study, the initial age was assumed to be 50 years, and the lifetime was extended to 80 years through 30 cycles. The cost was discounted at an annual rate of 5%. Since there is no established standard willingness to pay (WTP) threshold in China, we considered the one time of the 2021 China’s Gross Domestic Product (GDP) per capita at $12,044 per QALY as the benchmark value.

### Transition probability

Transition probabilities between different health states were taken from the literature review. As the treatment may achieve remission or relapse, the transition probability of active disease to remission or vice versa was obtained from the MENTOR trial, STARMEN trial and meta-analysis [17–19]. The MENTOR was a noninferiority trial conducted at 22 sites in the North America, which mainly focused on the complete or partial remission of proteinuria at 12 months with rituximab or cyclosporine in the treatment of IMN. The STARMEN was a clinical randomized controlled trial, which suggested that alternating treatment with corticosteroids and cyclophosphamide was superior to sequential treatment with tacrolimus and rituximab in primary membranous nephropathy. The meta-analysis comprehensively investigated the efficacy and safety of rituximab in patients with IMN by searching the MEDLINE, EMBASE and Cochrane Registry of Controlled Trials databases over the period of January 2000 to January 2020. The transition probability of active disease to ESRD or from remission to ESRD was derived from observational studies with long-term follow-up [7, 20–24]. The transition probability of ESRD to dialysis or to kidney transplant and probability of dialysis or kidney transplant to death were collected from the cohort studies in China [25, 26]. We assumed that the transition probability of active disease to death is equal to that of remission to death, and the data was from the chronic kidney disease mortality published in the official website of China National Bureau of Statistics [27]. All probabilities were applied to the model by converting to transition probabilities of one cycle (12 months). The calculation formula is as follow: r = -[In(1-P_1_)]/t_1_; P_2_ = 1-exp(-rt_2_); r represents the transient probability, and P_1_ and P_2_ represent the transition probability for a given cycle t_1_ and t_2_ respectively. Results are shown in Table 1.

**Table 1:** Base-case model variables and ranges used in a sensitivity analysis

Variable	Value	Range	Reference
**Transition Probabilities (12 months)**			
Probability of the composite of complete or partial remission by different drugs			
rituximab	0.60	0.4809–0.7190	[19]
cyclosporine	0.52	0.3985–0.6415	[17]
tacrolimus-rituximab	0.51	0.36–0.66	[18]
cyclophosphamide	0.79	0.67–0.90	[18]
Probability of relapse by different drugs			
rituximab	0.051	0.0353–0.667	[17]
cyclosporine	0.529	0.3612–0.6968	[17]
tacrolimus-rituximab	0.12	0.023–0.22	[18]
cyclophosphamide	0.027	0.01–0.06	[18]
Probability of remission to ESRD by different drugs			
rituximab	0.01	0–0.1	[20]
cyclosporine	0.023	0–0.1	[23]
tacrolimus-rituximab	0.0175	0–0.1	[25]
cyclophosphamide	0.023	0–0.1	[7]
Probability of active disease to ESRD	0.067	0–0.1	[7, 21, 22]
Probability of active disease or of remission to death			
50–60 years old	0.01	0–0.1	[27]
61–70 years old	0.016	0–0.1	[27]
71–80 years old	0.03	0–0.1	[27]
Probability of ESRD to kidney transplant	0.03	0–0.1	[25, 26]
Probability of dialysis to death	0.0422	0–0.1	[25, 26]
Probability of kidney transplant to death	0.017	0–0.1	[25, 26]
**Cost**			
price(specification/$)			
rituximab	100 mg/207.88	166.3–249.45	[28]
cyclosporine capsule	50 mg/0.93	0.75–1.12	[28]
tacrolimus capsule	1 mg/2.42	1.94–2.91	[28]
cyclophosphamide tablets	50 mg/0.88	0.66–0.99	[28]
methylprednisolone sodium succinate for injection	500 mg/17.96	14.36–21.55	[28]
prednisone tablets	5 mg/0.02	0.01–0.02	[28]
Other direct health care costs($)			
cost of dialysis	17,546.31/year	14,037–21,055	[26]
cost of kidney transplant	First year24,187.52 Subsequent year 17,249.1	13,799–30,698	[26]
cost of complete blood count (CBC)	7.58/time	6.06–9.09	[29]
cost of monitoring drug concentration	13.53/time	10.82–16.23	[29]
simple parenteral	2.97/time	2.37–3.56	[29]
inpatient stay	8.92/day	7.13–10.7	[29]
Costs related to adverse events($)			
minor infections	594.8	475.84–713.76	[29]
infusion-related reaction	52.04	41.63–62.45	[29]
pneumonia	594.8	475.84–713.76	[29]
gastrointestinal manifestation	1040.9	832.72–1249.08	[29]
leucopenia	297.4	237.92–356.88	[29]
Health utility values in each state			
active disease	0.746	0.6714–0.8206	[30]
remission	0.85	0.765–0.935	[30]
dialysis	0.689	0.6201–0.7579	[31]
kidney transplant	0.870	0.783–0.957	[31]
dead	0	0	

### Costs

In the model, all costs were converted into US dollars at an exchange rate of $1 = ¥6.7249. Direct costs referred to direct health care costs associated with the treatment of IMN, which covered drugs, treatment for drug-related adverse events (AEs), blood drug concentration monitoring, laboratory examination (the cost of complete blood count, CBC), hospital stays, dialysis, kidney transplant etc., as shown in Table 1. The drug price was taken from the public price from the Beijing Sunshine Drug Procurement Platform [28]. Drug-related AEs included minor infections, pneumonia, gastrointestinal manifestations, and infusion-related reaction, leucopenia. The incidence rate of drug-related AEs was extracted from clinical trials [17, 18] and shown in Table 2. Costs associated with blood drug concentration monitoring, examination, and hospital stays were obtained from the electronic healthcare records of a class A tertiary hospital in Beijing [29]. Costs of kidney transplant in the first year included surgery and nursing, laboratory and testing, immunosuppressive agents, accommodation and other costs. The surgery and nursing costs were excluded from the second year onwards [26]. Same cost is required for the drug concentration monitoring of cyclosporine and tacrolimus. The monitoring also takes place once per month and 12 times in a one-year cycle. Given that the costs were retrieved from literatures conducted in different time, plus taking into account of inflation rate, cost conversion was conducted so as to ensure that all the costs were comparable in the same time point. The conversion formula is as follow: cost multiplied by Consumer Price Index (CPI). For example, the cost for dialysis was $15,066 in 2013, and CPI from 2014 to 2021 were 102%, 101.4%, 102%,101.6%, 102.1%, 102.9%, 102.5%, 100.9% respectively, then costs for dialysis in 2021 was calculated to be $17,546.31 ($15,066 * 102% *101.4% *102% *101.6% *102.1% * 102.9% * 102.5% *100.9%).

**Table 2:** Probability of common adverse events associated with different drugs

Adverse events	cyclophosphamide(%)	cyclosporine(%)	rituximab(%)	tacrolimus-rituximab(%)	References
minor infections	31	15	18	23	[17–19]
infusion-related reaction	2	0	34	7	
pneumonia	7	9	2	3	
gastrointestinal manifestation	19	23	6	28	
leucopenia	38	0	0	3	

### Health utilities

QALYs was leveraged as the effectiveness measurement, which was calculated by multiplying the utility score by the time spent in a given state. Health state utility values were estimated on the basis of EQ-5D scale data from the previously published economic models in Thailand and Malaysia [30, 31]. For illustration, 1 represented for complete health and 0 for death, and the health utility values in each state were shown in Table 1.

### Sensitivity analysis

**Fig. 2:**
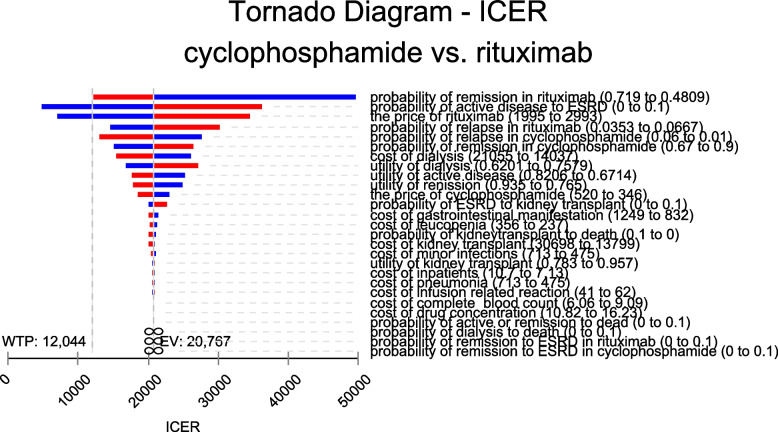
Tornado analysis (ICER) at a willingness-to-pay threshold of $12,044

We performed one-way sensitivity analysis to assess the impact of parameters on the outcome of model by varying transition probability parameters between lower and upper limits of 95% confidence interval (some transition probabilities were assumed as 0 to 0.1 in range for lacking sufficient rationale), the costs within ± 20% of its baseline value, the utilities within ± 10%, and other parameters were unchanged. The outcome was displayed in tornado diagrams. The variables that have the greatest impact on ICER estimate were shown in Fig. 2.

Probabilistic sensitivity analysis (PSA) was conducted using 1000 iterations of Monte-Carlo simulation by simultaneous and random preset variations of parameters to evaluate the optimal strategies under different hypothetical WTPs ($12,044 and $36,134). Gamma distribution was used to represent the uncertainty in costs. Beta distribution was used to represent the uncertainty in utility and transition probability. Uncertainty was displayed in a scatter plot and cost-effectiveness acceptability curve (CEAC).

## Results

### Base-case analysis

The total costs of cyclophosphamide, cyclosporine, rituximab and tacrolimus-rituximab for progressive IMN patients over lifetime were $65,524.19, $93,861.29, $74,686.39, and $93,848.33 respectively, in correspondence to QALY values of 19.09, 17.46, 19.53 and 18.62 life years. Compared with cyclophosphamide, the incremental costs were $28,337.09 and $28,324.13, and incremental QALYs is 1.63 and -0.47 for cyclosporine and tacrolimus-rituximab, respectively. Both cyclosporine and tacrolimus-rituximab incurred incremental costs but showed reduced QALYs, which were considered as strictly dominated. In comparison with cyclophosphamide, rituximab provided an additional 0.44 QALYs at an additional cost of $9,162.19, resulting in an ICER of $20,767 per QALY. Hence, the ICER of rituximab exceeded the WTP threshold of $12,044 per QALY in China, which was deemed as not cost-effective and unaffordable for the Chinese payers as shown in Table 3.

**Table 3:** Summary of cost ($) and outcome results in the base-case analysis

Drug	Utility (QALY)	Increased utility	Cost($)	Increases costs	ICER
cyclophosphamide	19.09	0	65,524.19	0	0
cyclosporine	17.46	-1.63	93,861.29	28,337.09	-17,337(dominated)
rituximab	19.53	0.44	74,686.39	9,162.19	20,767
tacrolimus-rituximab	18.62	-0.47	93,848.33	28,324.13	-60,829(dominated)

Dominated: high cost and low effective

### Sensitivity analysis

One-way sensitivity analysis was performed to further examine the robustness of the ICER results comparing rituximab and cyclophosphamide. As shown in Fig. 2, top five most influential parameters included the transition probability of remission in rituximab, the transition probability of active disease to ESRD, the price of rituximab, the transition probability of relapse in rituximab, the transition probability of relapse in cyclophosphamide. The ICER of rituximab was less than WTP threshold when probability of active disease to ESRD smaller than 0.036 and when the price of rituximab below $181/100 mg. The model was robust to variance in all the other parameters inputs.

**Fig. 3:**
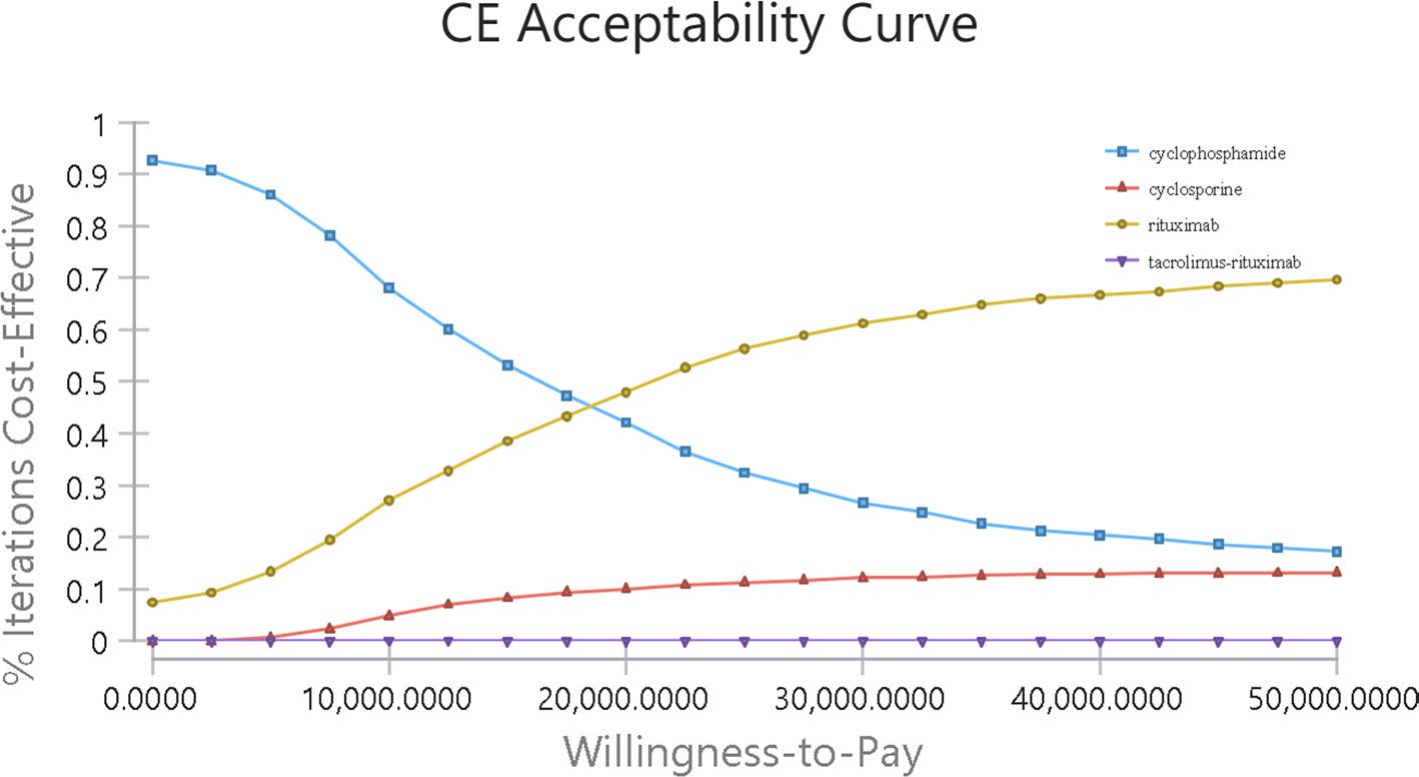
Cost-effectiveness acceptability curve for four drugs

Probabilistic sensitivity analysis (PSA) was displayed by CEAC in Fig. 3. When setting the WTP value to $12,044, the acceptable probability of cyclophosphamide was 62.4%. The acceptable probability of cyclosporine and rituximab increased with a higher WTP value was adopted. Regardless of the WTP change, the acceptable probability of tacrolimus-rituximab remained zero. When WTP value exceeded $18,300, the acceptable probability of rituximab was greater than that of cyclophosphamide.

**Fig. 4:**
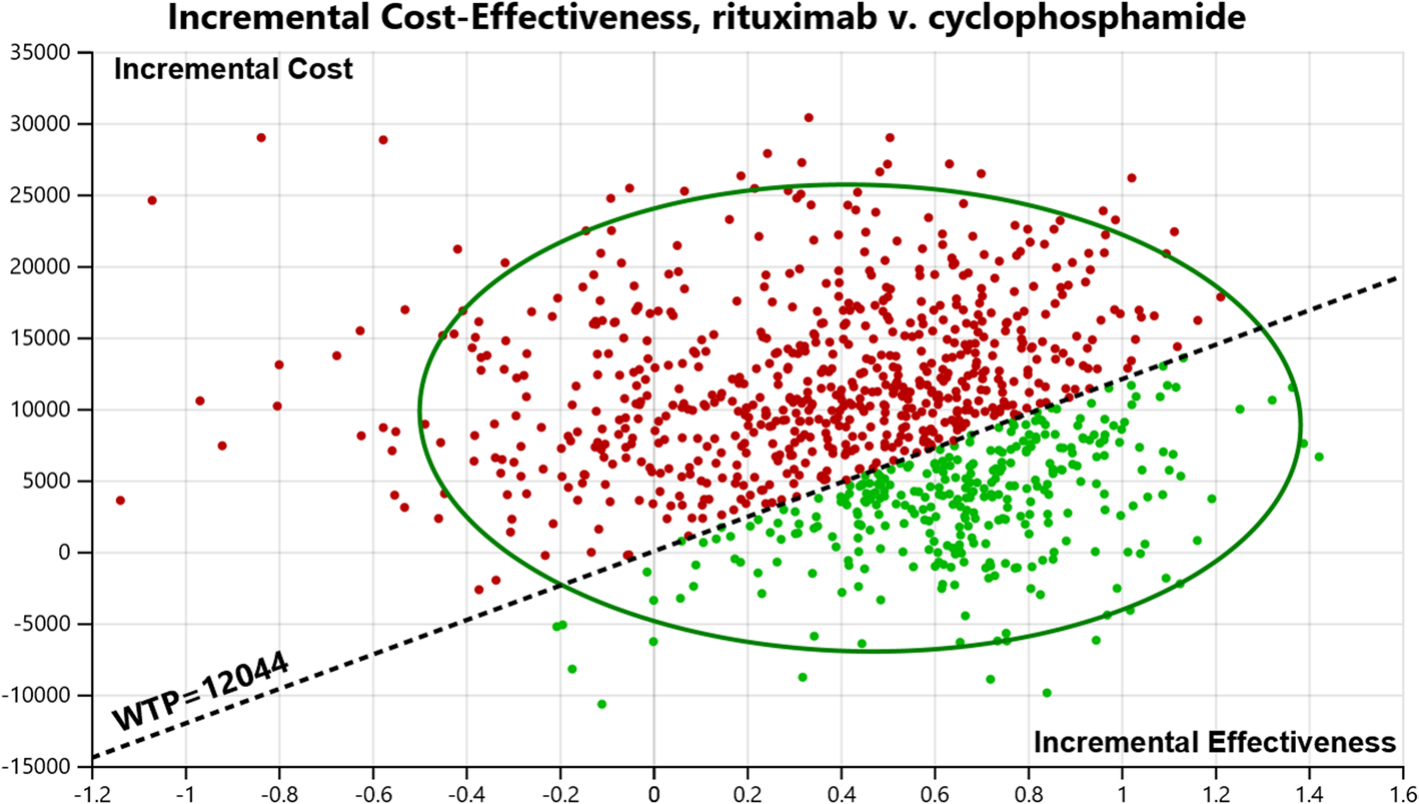
Incremental cost-effectiveness scatter plot of probabilistic sensitivity analysis (the slope of WTP $12,044/QALY)

The ICER scatter plots in Fig. 4 reflected the changes and concentrations of the ICER values in the PSA. And 79.7% of scatter plots indicated that rituximab gained more QALYs than cyclophosphamide but with higher cost. At a WTP threshold of $12,044 per QALY, rituximab was considered cost-effective in 32.6% of the simulations. At a WTP threshold of $36,134 per QALY, rituximab was assessed as cost-effective in 69.4% of the simulations as shown in Supplementary Fig. S2.

## Discussion

This study conducted a pharmacoeconomic evaluation for the four treatment regimens in a designated cohort of IMN patients above 50 years over a lifetime horizon. To our knowledge, this is the first cost-effectiveness analysis to assess cyclophosphamide, cyclosporine, rituximab, tacrolimus-rituximab for the treatment of IMN in China. In the base case analysis, the ICER of cyclosporine and tacrolimus-rituximab was negative (-17,337, -60,829), which were considered strictly dominated. In the comparison between cyclophosphamide and rituximab, when WTP threshold value of per QALY gained reaching $12,044, cyclophosphamide was the best choice in treating IMN. In the PSA, as shown in the CEAC, the acceptable probability of rituximab was increased with a rise in WTP. We considered rituximab as cost-effective compared with cyclophosphamide when assuming a WTP threshold of $18,300 per QALY in the exploratory analysis of the Markov model.

Our research results provided important insights and innovations that were not discussed in previous literature. A network meta-analysis and a cost-effectiveness analysis by Dai et al. [32] from the Chinese perspective found that cyclophosphamide was the cheapest treatment with a curative potential for IMN patients, which was similar to our finding. However, Dai’s study only introduced two exclusive health states (health and disease) and lacked a consideration for the states in ESRD phase. Meanwhile, Dai et al. took into account only the cost of medication, while discarding the costs of medication monitoring and of treating adverse reactions. To compensate the limitations of previous studies, we conducted a more comprehensive analysis by taking into account of all possible health states and cost components. In our viewpoint, cyclophosphamide’s high remission rate and lower price were two contributing factors that made our study and Dai’s study reach the similar conclusion. However, contrary results to our findings were showed in another study by Hamilton et al. [33], in which a decision-analytic model was developed to estimate the cost-effectiveness of rituximab versus cyclophosphamide from the perspective of the National Health Service in UK. They suggested that, over a lifetime horizon, rituximab remained a cheaper option with an incremental cost of -£5,251.03 but with a reduced QALY of -0.512, resulting in an ICER of £10,246.09. The possible reason for the divergent results might be attributed to the high cost of simple parenteral in UK, which added cost to the inexpensive cyclophosphamide regimen. Considering that the price of simple parenteral in UK was about 100 times of the price in China, it is unsurprising to see that cyclophosphamide was cost-effectiveness than rituximab in our study, but not in study by Hamilton et al. For the difference in QALY, in Hamilton’s study, the reduction of QALY for rituximab over time is partly associated with the slightly increasing risk of death. In our study, the mortality rate was assumed to be the same. In short, the economic evaluation needs to consider local medical costs and patient’s health utility.

The one-way sensitivity analysis revealed that the results of the model were most sensitive to the probability of remission in rituximab, and other influential parameters included the probability of active disease to ESRD and the price of rituximab. For the probability of active disease to ESRD, we assumed its range as 0 to 0.1. We found that rituximab became more favorable when the probability value was less than 0.036. In other words, rituximab has more cost-effectiveness for patients at relatively low risk of developing ESRD [34, 35]. As for the price, rituximab became affordable as its price decreased, as demonstrated in that the ICER of rituximab was less than WTP threshold value when the price of rituximab was reduced to $181/100 mg. It seems reasonable to predict that, in the long run, rituximab is likely to become a better choice if it is included in centralized medicine procurement program. From PSA analysis, there is a possibility that rituximab will gain more advantages with the increase of GDP per capita in the future.

The following findings from clinical studies may provide some explanations for the disadvantage of cyclosporine and tacrolimus-rituximab: (1) CNIs takes effect quickly in the treatment of IMN, but its remission rate is lower than that of cyclophosphamide [36, 37]; (2) Patients with renal insufficiency is more prone to have renal progression in cyclosporine group than in cyclophosphamide group given the higher risk of drug renal toxicity after cyclosporine treatment [38].

### Limitations

As with any model, there are limitations to our analysis. First, the parameters for treatment effectiveness in this study were derived from RCT or cohort studies. These studies are drug efficacy studies and did not take into account the effect of patient compliance on treatment outcomes. Therefore, parameters from these studies might lead to a certain bias in the result of our model. At the same time, we didn’t find any studies about the effect of patient compliance on treatment outcomes in IMN patients. Besides, the model assumes that the transition probability was a constant value, but it always changes with time in the real-world setting. Given the uncertainty in the model, this highlights the importance of conducting high quality, long-term prospective research comparing these four regimens in the future. Second, another limitation involves the definition and collecting of cost data. This study only calculated the direct medical costs, and did not consider the indirect costs (loss of work expenses, escort expenses, and etc.) and hidden costs deriving from patient acceptance and patient compliance. These costs are challenging to collect and monetized, but it is generally accepted that this impact is marginal for cost-effectiveness analysis. In addition, this study conducted a sensitivity analysis by varying the costs of four treatment regimens and did not find any difference. Third, the health utility value obtained from the published literature could not accurately reflect the clinical effect on Chinese IMN patients. In fact, there will be differences in health utility values between countries for the sake of economic and cultural reasons [39]. However, the sensitivity analysis results of this study suggest that this indicator has little effect on the results. Fourth, Markov model simplified the whole process of IMN disease and did not include complications caused by IMN (such as thrombus of lower extremity veins), which will introduce bias in results.

## Conclusion

In conclusion, our results indicated that cyclophosphamide could be considered as a more cost-effective strategy for progressive IMN in China. Although the ICER of rituximab exceeds the WTP threshold value, it has a slight improvement in QALYs. Further evidence is needed using data from large-scale studies. Therefore, the doctor should choose the appropriate treatment regimen with a thorough consideration of the patient’s disease and economic condition.

## Supplementary Information

**Additional file 1: Fig. S1.** Tree diagram of the Markov model. **Fig. S2.** Incremental cost-effectiveness scatter plots of probabilistic sensitivity analysis (the slope of WTP $36,134/QALY).

## Data Availability

All data generated or analysed during this study are included in this published article.
