# Supplementary material for: Immunosuppressive therapy for progressive idiopathic membranous nephropathy: a cost-effectiveness analysis in China
Source: BMC Health Serv Res. 2023 Apr 12;23:361. doi: 10.1186/s12913-023-09365-z (PMC10091593; doi:10.1186/s12913-023-09365-z)
Supplement: Supplementary file 1 — Additional file 1: Fig. S1. Tree diagram of the Markov model. Fig. S2. Incremental cost-effectiveness scatter plots of probabilistic sensitivity analysis (the slope of WTP $36,134/QALY). [file 12913_2023_9365_MOESM1_ESM.pdf]

Supplementary Materials

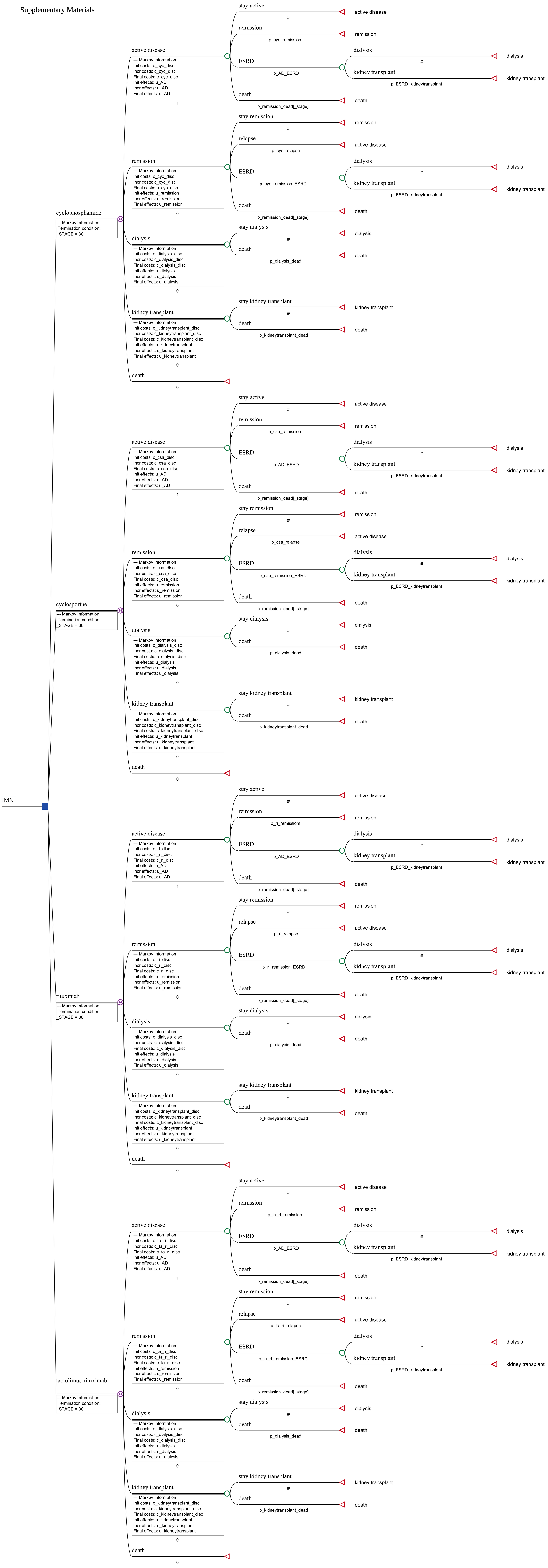

Fig.S1 Tree diagram of the Markov model

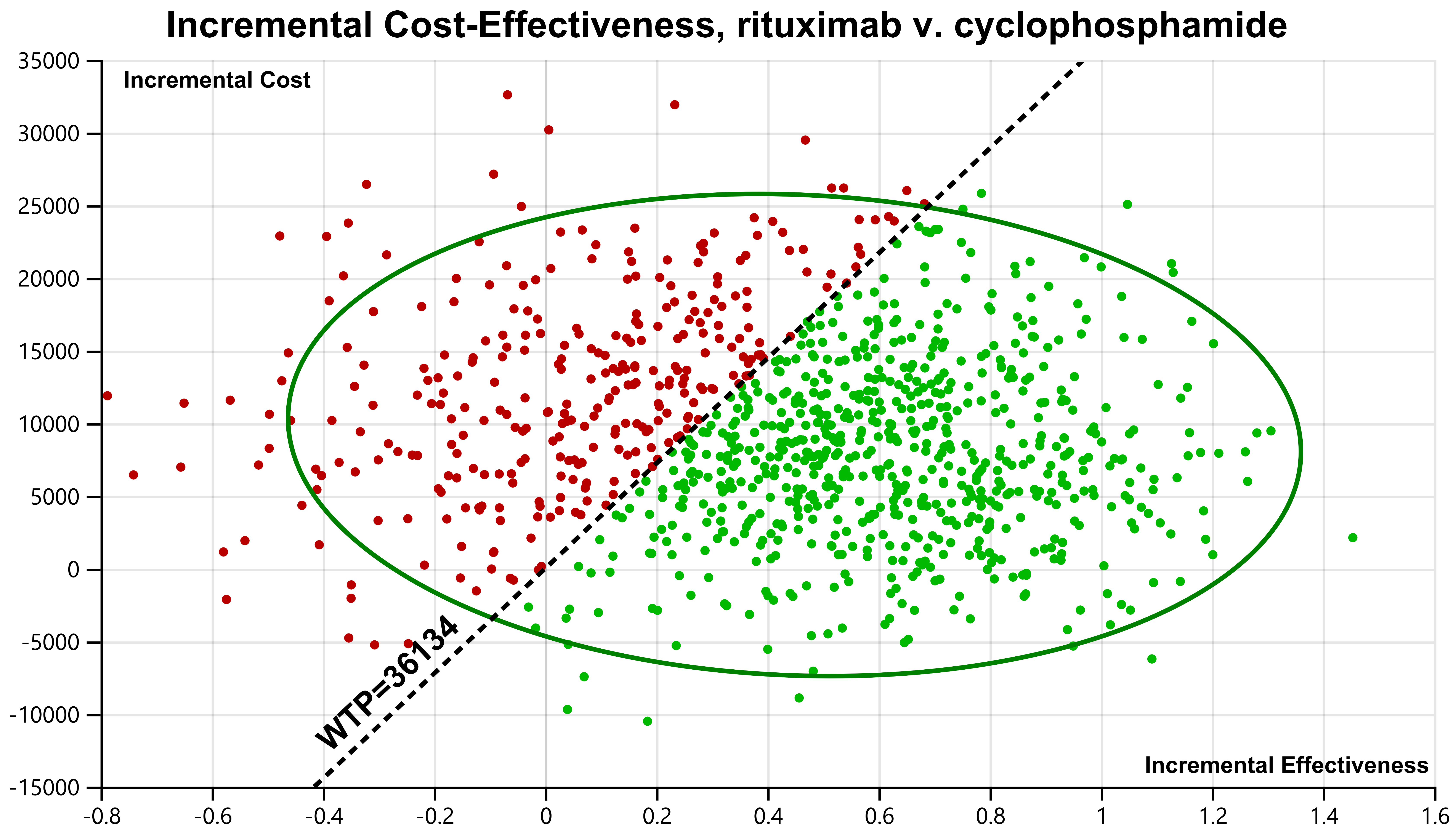

Fig.S2 Incremental cost-effectiveness scatter plot of probabilistic sensitivity analysis (the slope of WTP $36134/QALY)
